# Oral streptococci subvert the host innate immune response through hydrogen peroxide

**DOI:** 10.1038/s41598-021-04562-4

**Published:** 2022-01-13

**Authors:** Yi Ling Tang, Tiow Suan Sim, Kai Soo Tan

**Affiliations:** 1 Faculty of Dentistry, National University of Singapore, Singapore, Singapore; 2 Department of Microbiology and Immunology, Yong Loo Lin School of Medicine, National University of Singapore, Singapore, Singapore

**Keywords:** Microbiology, Diseases, Pathogenesis

## Abstract

In periodontal health, oral streptococci constitute up to 80% of the plaque biofilm. Yet, destructive inflammatory events of the periodontium are rare. This observation suggests that oral streptococci may possess mechanisms to co-exist with the host. However, the mechanisms employed by oral streptococci to modulate the innate immune response have not been well studied. One of the key virulence factors produced by oral streptococci is hydrogen peroxide (H_2_O_2_). In mammalian cells, H_2_O_2_ triggers the activation of nuclear factor erythroid 2-related factor 2 (Nrf2), a key pathway mediating antioxidant defence. This study aimed to determine (1) if H_2_O_2_ producing oral streptococci activated the Nrf2 pathway in macrophages, and (2) if the activation of Nrf2 influenced the innate immune response. We found that oral streptococci downregulated the innate immune response in a H_2_O_2_ dependent manner through the activation of the Nrf2. The activation of the Nrf2 signalling pathway led to the inhibition of nuclear factor kappa-light-chain-enhancer of activated B cells (NFĸB), the key transcription factor regulating pro-inflammatory response. This study showed for the first time that oral streptococci are unlikely passive bystanders but could play an active role in the maintenance of periodontal health by preventing overt inflammation.

## Introduction

Periodontal disease is a plaque biofilm-mediated chronic inflammatory disease that affects the supporting structure of the teeth. Periodontitis affects 40% of the adult population and is the most common cause of tooth loss globally^1^. Patients suffering from periodontitis are also at higher risk for systemic diseases such as diabetes^2^ and cardiovascular disease^3^. The oral cavity is heavily colonised by microbial communities, with more than 700 bacterial species identified^4,5^. Collectively referred to as the oral microbiome, these bacterial species colonise distinct niches of the oral cavity, forming biofilms. Disturbances in the composition of plaque biofilm are the primary aetiological factor for the initiation of periodontal disease^6^.

Oral streptococci are the pioneer colonisers of the plaque biofilm. In periodontal health, oral streptococci are the dominant species of oral microbiota, which constitute between 60 and 80% of the total cultivable microbial flora^7–10^. Although the gingiva is exposed to large amounts of oral streptococci, destructive inflammation is rare, suggesting that these bacterial species likely possess mechanisms that modulate the innate immune response, facilitating co-existence with the host. However, mechanisms that enable oral streptococci to achieve symbiosis with the host have not been well studied.

The mitis group streptococci produce hydrogen peroxide (H_2_O_2_) as a by-product of aerobic metabolism^11^. Three enzymatic pathways for the generation of H_2_O_2_ in oral streptococci have been described. Pyruvate oxidase, encoded by the *spxB* gene is highly conserved in oral streptococci. This enzyme catalyses the conversion of inorganic phosphate and pyruvate to acetyl phosphate, carbon dioxide and H_2_O_2_^12^_._ Oral streptococci also produce H_2_O_2_ via lactate and L-amino acid oxidases. Lactate oxidase catalyses the formation of H_2_O_2_ and pyruvate from lactate and oxygen, while L-amino acid oxidase catalyses the formation of peptone and H_2_O_2_ from L-amino acid in the presence of oxygen and water^13,14^.

The production of H_2_O_2_ by oral streptococci is thought to confer a competitive advantage over pathogenic microbes, enhancing their colonisation and survival in the oral cavity. Studies have reported that the production of H_2_O_2_ secreted by these organisms inhibited the growth of oral pathogens such as *Streptococcus mutans*^15^, and *Porphorymonas gingivalis*^16^. Thus, it has been put forth that H_2_O_2_ production likely contributes to shaping the biofilm composition towards a microbiome compatible with health. However, it is currently unknown if H_2_O_2_ secreted by oral streptococci modulates the innate immune response.

H_2_O_2_ belongs to the family of molecules termed reactive oxygen species (ROS). High concentrations of H_2_O_2_ elicit cell damage and cell death. At non-cytotoxic concentrations, H_2_O_2_ is an important intracellular signalling molecule that controls cellular processes including inflammation^17–19^. However, there have been conflicting reports concerning the pro- and anti-inflammatory properties of H_2_O_2_. For instance, H_2_O_2_ was demonstrated to stimulate the production of the pro-inflammatory cytokine tumour necrosis factor- α (TNF-α) in macrophages through the activation of nuclear factor kappa-light-chain-enhancer of activated B cells (NFĸB) signalling pathway^20,21^. However, H_2_O_2_ secreted by *Streptococcus pneumoniae* was shown to prevent the activation of inflammasome in macrophages, consequently downregulating the innate immune response^22^.

The key cellular signalling pathway activated by H_2_O_2_ in mammalian cells is the nuclear factor E2-related factor 2 (Nrf2) antioxidant pathway. Nrf2 belongs to the Cap ‘n’ Collar (CNC) subfamily of basic region leucine zipper (bZaip) transcription factors. It is a ubiquitous transcription factor that regulates the expression of an array of antioxidant and detoxification genes, controlling the pathophysiological and physiological outcomes during oxidative stress^23^. Under normal physiological conditions, the expression of Nrf2 is minimal, due to its rapid turnover through constant degradation by the ubiquitin–proteasome system. During oxidative stress, the activation of Nrf2 pathway causes the nuclear translocation of Nrf2, inducing the expression of genes harbouring the antioxidant response element (ARE), a cis-acting DNA enhancer motif located in the promoter region of numerous antioxidant genes and enzymes that metabolise xenobiotics^24,25^. In addition, the Nrf2 signalling pathway appears to also play critical roles in regulating inflammation. For instance, Nrf2 knockout animals showed exacerbation of inflammation in emphysema and during sepsis, and have a predisposition to develop autoimmune and inflammatory phenotypes in various tissues^26–29^.

It is currently unknown if oral streptococci regulate the Nrf2 pathway. The objectives of this study were, (1) to determine if H_2_O_2_ producing oral streptococci activated the Nrf2 signalling pathway in macrophages, and (2) to determine if the activation of the Nrf2 pathway influenced the innate immune response in macrophages. We hypothesised that H_2_O_2_ secreted by oral streptococci upregulates the Nrf2 signalling pathway, dampening the innate immune response, thus enabling the maintenance of host-microbe homeostasis to sustain periodontal health.

## Results

### H_2_O_2_ producing oral streptococci activated the Nrf2-ARE pathway

**Figure 1:**
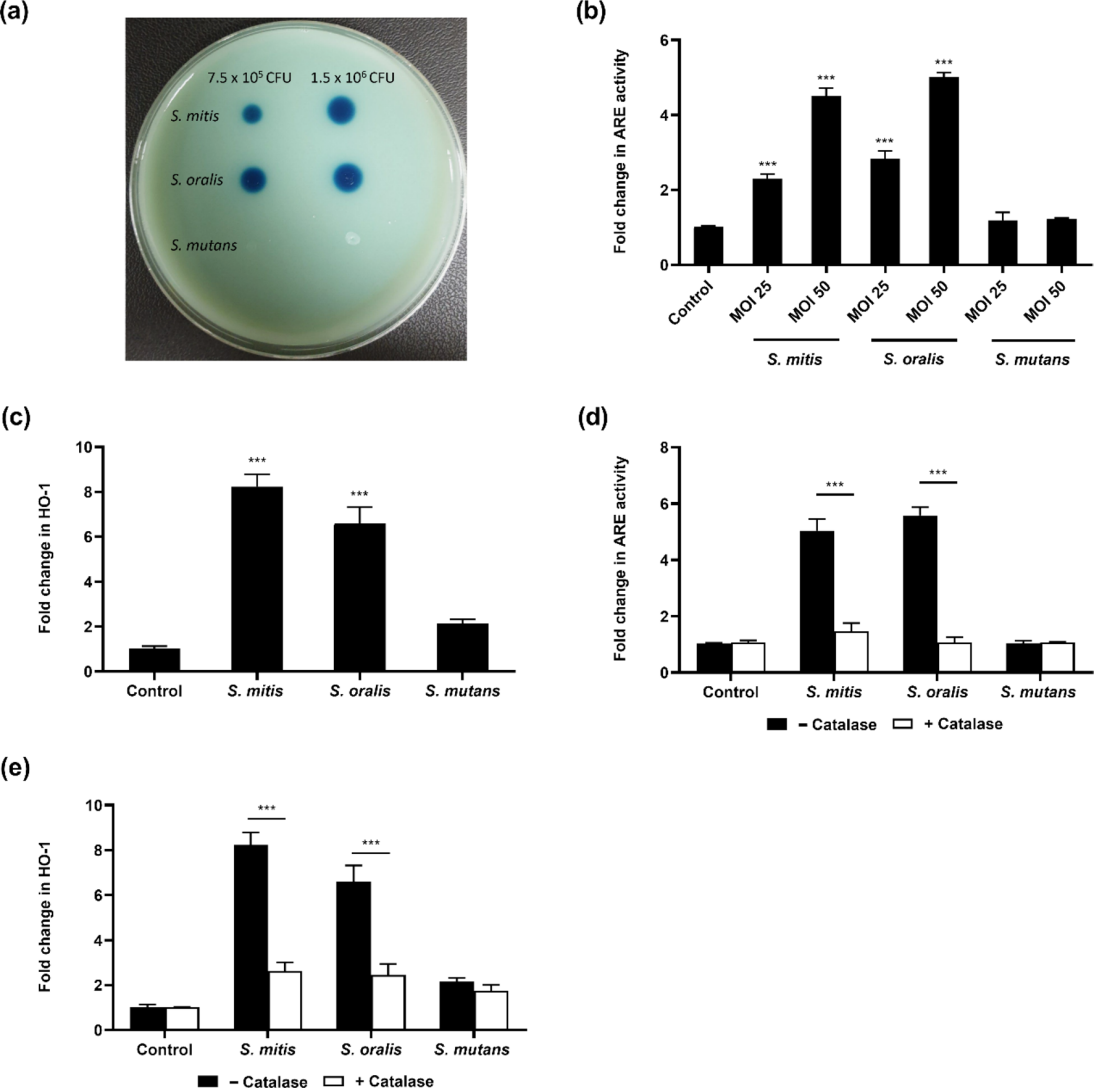
H_2_O_2_ producing oral streptococci activated the Nrf2-ARE pathway. (**a**) The amount of H_2_O_2_ produced by oral streptococci was determined by the formation of blue halos on Prussian blue agar plates. (**b**) Raw 264.7 cells stably expressing the ARE-SEAP reporter were infected with *S. mitis*, *S. oralis* or *S. mutans* at the indicated MOIs. Cellular Nrf2-ARE activity was quantified by SEAP reporter assay. The expression of (**c**) HO-1 were determined by qRT-PCR. Raw 264.7 cells stably expressing the ARE-SEAP reporter were infected with *S. mitis* or *S. oralis* at MOI 50:1 in the presence and absence of 25U/mL of catalase. (**d**) ARE activity was determined by SEAP reporter assay while the expression of (**e**) HO-1 were determined by qRT-PCR. ***p < 0.001 compared to the respective control group.

**Figure 2:**
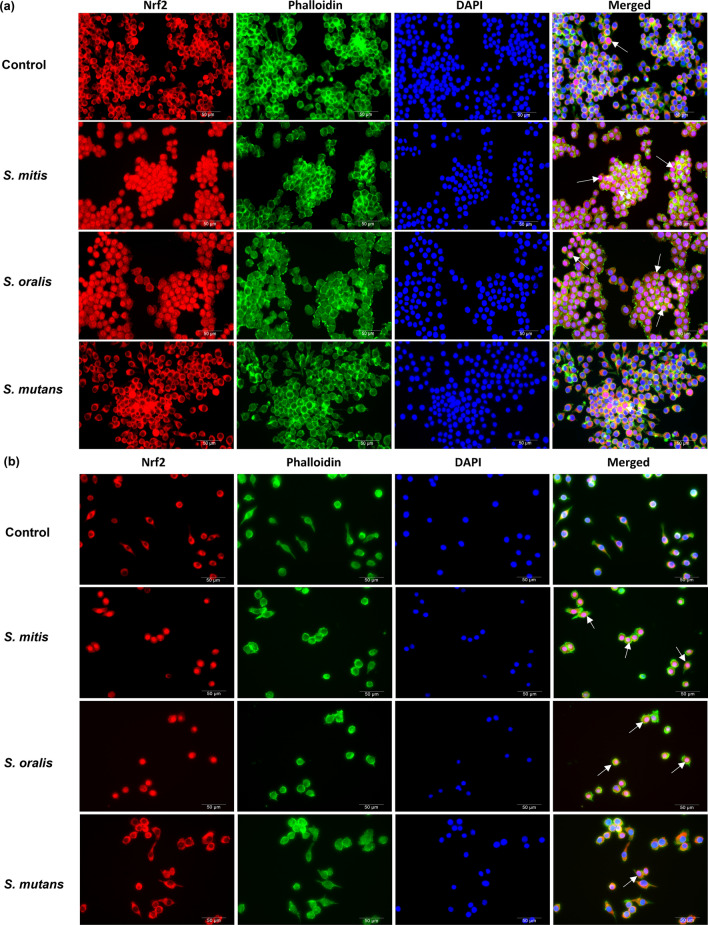
H_2_O_2_ producing oral streptococci induced nuclear translocation of Nrf2. (**a**) Raw 264.7 cells and (**b**) BMDMs were infected with the indicated oral streptococci species at MOI 50:1 for 5 h. Cellular localisation of Nrf2 was visualised following staining with anti-Nrf2 antibody (red). Cells were also stained with phalloidin (green), and DAPI (blue) for visualisation of cytoplasm and nuclei respectively. Representative immunofluorescence images are shown at 400× magnification. White arrows indicate nuclear translocated Nrf2. Scale bar: 50 μm.

To determine if H_2_O_2_ producing oral streptococci modulated the Nrf2 signalling pathway, Raw 264.7 cells stably expressing the ARE-SEAP reporter were infected with H_2_O_2_ producing (*S. mitis* and *S. oralis*), and H_2_O_2_ non-producing oral streptococci species (*S. mutans*) (Fig. 1a). *S. mitis* and *S. oralis* but not *S. mutans* elicited a dose-dependent increase in Nrf2-ARE activity (Fig. 1b). The expression of the antioxidant genes namely, heme oxygenase-1 (HO-1) (Fig. 1c) and glutathione Peroxidase-3 (GPx-3) (Supplementary Fig. 1a) corroborated with the findings of the Nrf2-ARE reporter assay where *S. mitis* and *S. oralis* significantly activated the expression of these genes but not *S. mutans*. When exogenous catalase was added, *S. mitis* and *S. oralis* mediated activation of ARE (Fig. 1d) and upregulation of the antioxidant genes were abrogated (Fig. 1e; Supplementary Fig. 1b). In addition, it was observed that Nrf2 was predominantly sequestered in the cytoplasm in the control untreated cells (Fig. 2). However, treatment of Raw 264.7 macrophages with *S. mitis* or *S. oralis* led to the nuclear translocation of Nrf2, which was not observed in *S. mutans* infected cells (Fig. 2a). Similar results were obtained when bone marrow derived macrophages (BMDMs) were infected with these oral streptococci species (Fig. 2b). Both trypan blue exclusion and LDH assays showed that bacterial infection did not significantly affect the viability of macrophages (Supplementary Fig. 2). Collectively, these results demonstrated that *S. mitis* and *S. oralis* elicited the activation of antioxidant defence signalling in macrophages in a H_2_O_2_ dependent manner.

### Bacterial pyruvate oxidase mediated the activation of Nrf2 pathway in macrophages

**Figure 3:**
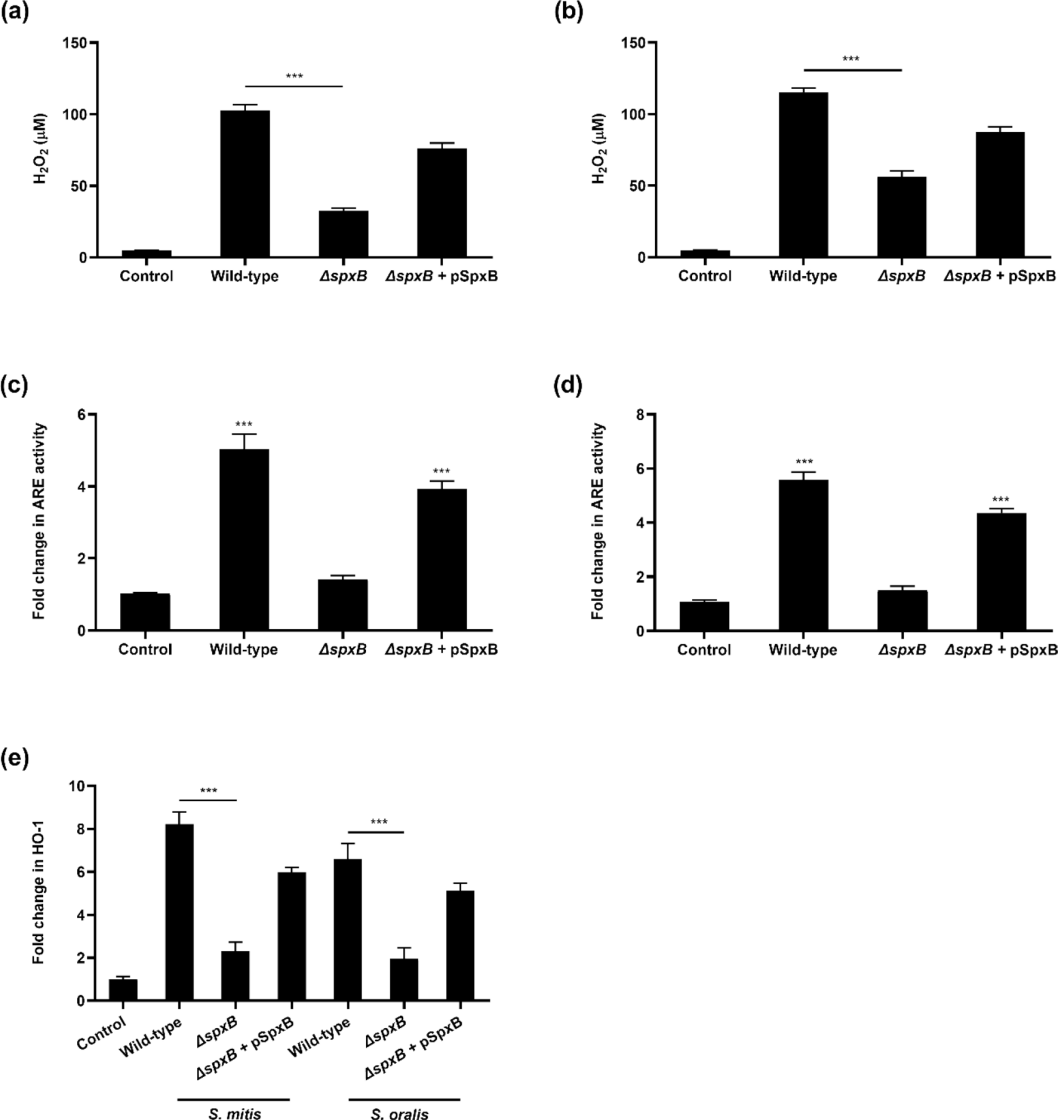
Bacterial SpxB mediated the activation of Nrf2-ARE. The amount of H_2_O_2_ produced by wild-type, *ΔspxB* mutants and *ΔspxB* complemented mutant (*ΔspxB* + pSpxB) of (**a**) *S. mitis* and (**b**) *S. oralis* were measured. Raw 264.7 cells stably expressing the Nrf2-ARE SEAP reporter were infected with wild-type, *ΔspxB* mutants or *ΔspxB* complemented mutants of (**c**) *S. mitis* or (**d**) *S. oralis*. Cellular ARE activity was quantified by SEAP reporter assay. The expression of (**e**) HO-1 was determined by qRT-PCR. ***p < 0.001 compared to the control group.

One of the key enzymes involved in the synthesis of H_2_O_2_ by oral streptococci is pyruvate oxidase (SpxB)^11,12^. To determine whether SpxB was the bacterial effector responsible for the activation of Nrf2, *ΔspxB* mutants of *S. mitis* and *S. oralis* were genetically engineered. Compared to the respective wild-type strains, *ΔspxB* mutants demonstrated 2–3 folds reduction in H_2_O_2_ production (Fig. 3a,b), which corresponded with reduced Nrf2-ARE activation (Fig. 3c,d) and the expression of HO-1 and GPx-3, compared to their respective wild-type strains (Fig. 3e and Supplementary Fig. 3). Restoration of SpxB function in *S. mitis* and *S. oralis ΔspxB* mutants through complementation restored the mutants’ ability to produce H_2_O_2_ which led to the activation of the host Nrf2 antioxidant defence system (Fig. 3 and Supplementary Fig. 3). These results provide evidence that SpxB mediated H_2_O_2_ production was responsible for the activation of the Nrf2 pathway, and the induction of synthesis of antioxidant genes in macrophages.

### Bacterial secreted H_2_O_2_ inhibited NFĸB

**Figure 4:**
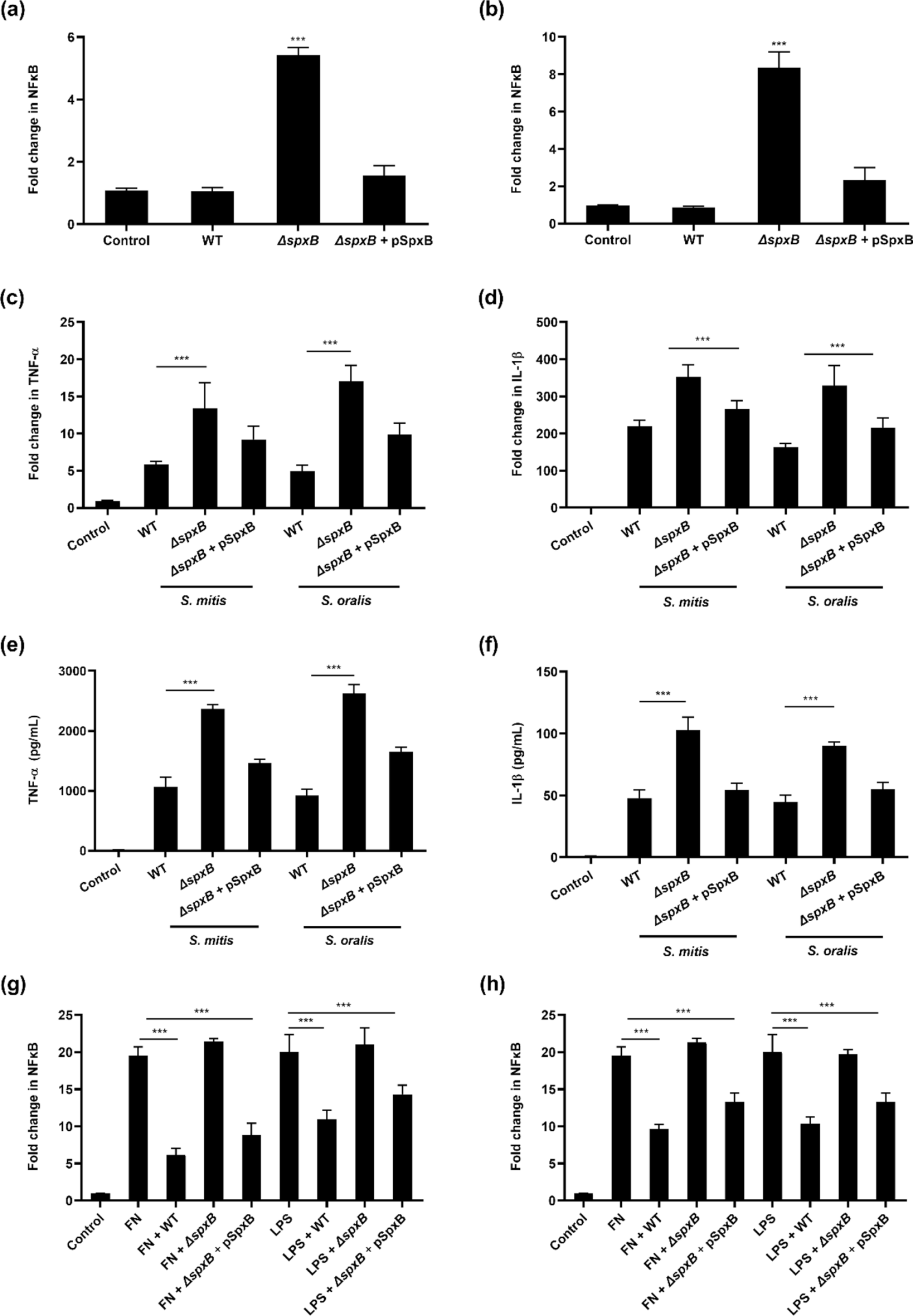
Bacterial pyruvate oxidase mediated suppression of the host NFĸB. Raw 264.7 cells stably transfected with the NFĸB SEAP reporter were infected with wild-type (WT) bacteria, *ΔspxB* mutants and *ΔspxB* complemented mutant (*ΔspxB* + pSpxB) of (**a**) *S. mitis* or (**b**) *S. oralis*. Cellular NFĸB activity was quantified by SEAP reporter assay. ***p < *0.001* compared to the control group. The expression of (**c**) TNF-α and (**d**) IL-1β were determined by qRT-PCR. Amount of (**e**) TNF-α and (**f**) IL-1β protein was determined by ELISA. Macrophages were incubated with WT, *ΔspxB* mutants or *ΔspxB* complemented mutant of (**g**) *S. mitis* or (**h**) *S. oralis,* in the presence or absence of *F. nucleatum* (FN) and LPS. ***p < 0.001*.*

To determine whether H_2_O_2_ secreted by oral streptococci modulated the innate immune response, Raw 264.7 cells stably expressing the NFĸB-SEAP reporter were infected with wild-type or the *ΔspxB* mutants of *S. mitis* and *S. oralis*. Interestingly, macrophages infected with *ΔspxB* mutants demonstrated significantly higher levels of NFĸB activation compared to their respective wild-type strains (Fig. 4a,b). Similarly, the expression of the proinflammatory cytokines i.e. TNF-α and IL-1β by macrophages infected with *ΔspxB* mutants were higher compared to cells infected with wild-type strains at the mRNA (Fig. 4c,d) and protein levels (Fig. 4e,f). Complementation of SpxB function in *S. mitis* and *S. oralis ΔspxB* mutants restored the *ΔspxB* mutants’ ability to produce H_2_O_2_, and suppressed NFĸB signalling pathway and synthesis of the proinflammatory cytokines (Fig. 4a–f). Furthermore, H_2_O_2_ secreted by *S. mitis* and *S. oralis* suppressed *Fusobacterium nucleatum* or lipopolysaccharide (LPS)-induced NFĸB (Fig. 4g,h). Collectively, these results demonstrated a novel role of SpxB in immune suppression.

### Activation of Nrf2-ARE by oral streptococci H_2_O_2_ inhibited the NFĸB signalling pathway

**Figure 5:**
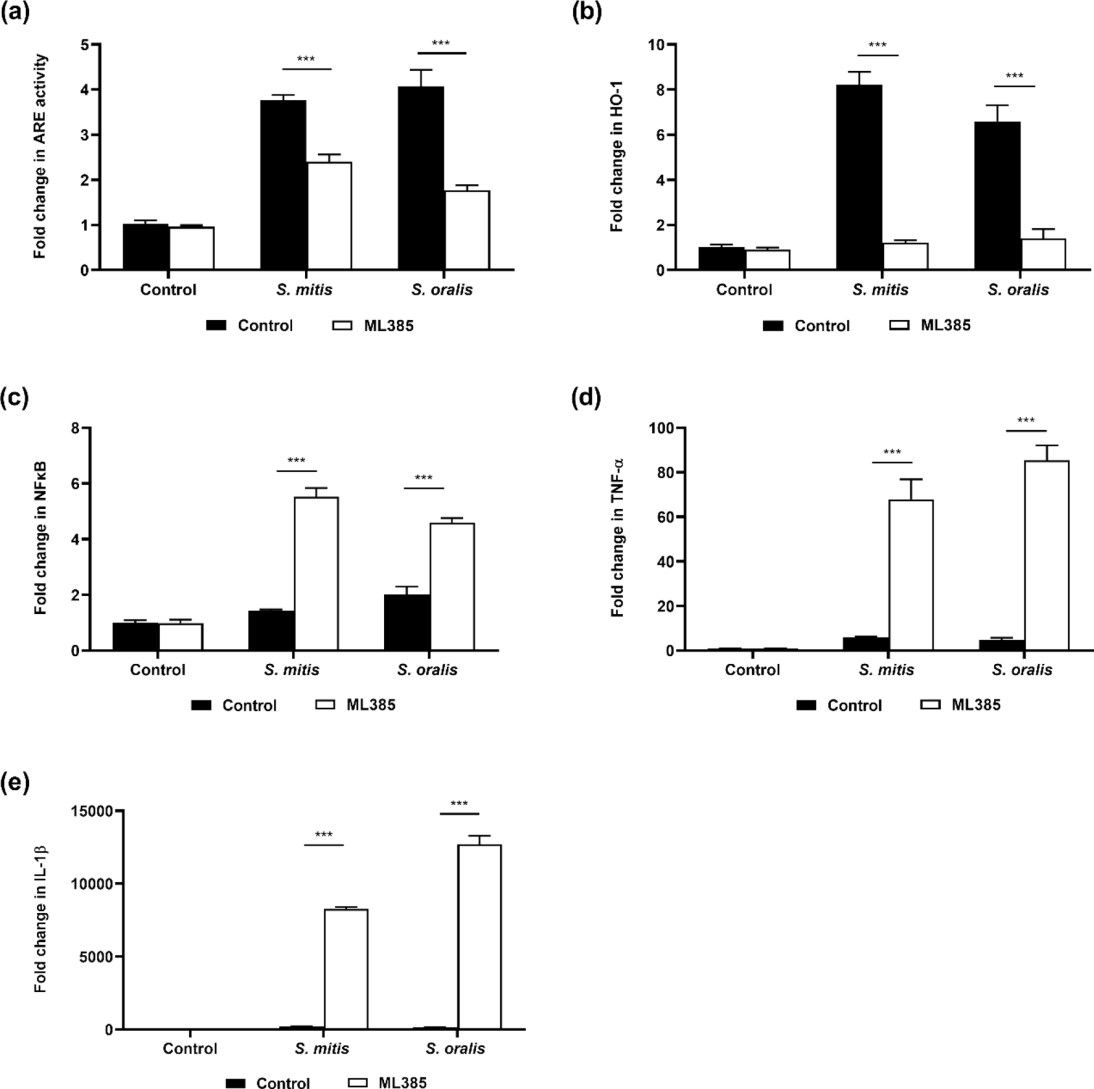
Activation of Nrf2 by oral streptococci inhibited the NFĸB signalling pathway. Raw 264.7 cells were pre-treated with 10 µM ML385 for 24 h prior to infection with oral streptococci. (**a**) Raw 264.7 cells stably transfected with the ARE SEAP reporter were infected with *S. mitis* or *S. oralis*. The expression of (**b**) HO-1 was determined by qRT-PCR. (**c**) Raw 264.7 cells stably transfected with the NFĸB SEAP were infected with bacteria. The expression of (**d**) TNF-α and (**e**) IL-1β were determined by qRT-PCR. ***p < 0.001*.*

To determine if the activation of the host antioxidant pathway could potentially inhibit the innate immune response of macrophages, Raw 264.7 cells were treated with ML385, a Nrf2 inhibitor. It was observed that treatment of macrophages with ML385 led to the inhibition of *S. mitis* or *S. oralis* mediated Nrf2-ARE activation (Fig. 5a), and downregulation of the expression of HO-1 and GPx-3 (Fig. 5b, Supplementary Fig. 4). However, the inhibition of Nrf2 pathway led to enhanced NFĸB activation and potentiation of TNF-α and IL-1β production (Fig. 5c–e). ML385 did not affect the viability of the macrophages and bacteria (Supplementary Fig. 5).

**Figure 6:**
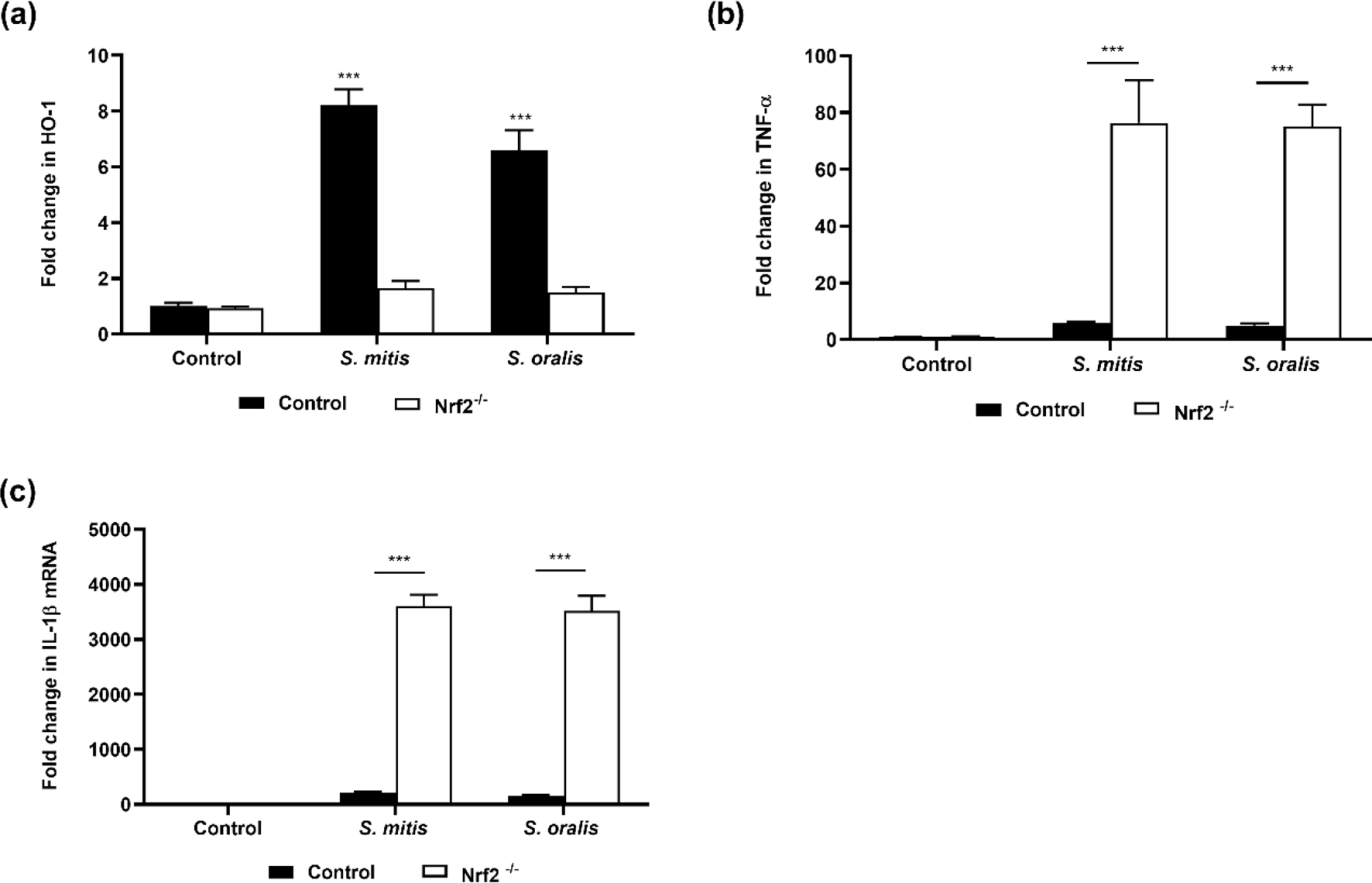
Activation of Nrf2 inhibits the innate immune response. Wild-type (control) or Nrf2^-/-^ Raw 264.7 cells were infected with oral streptococci for 8 h and the expression of (**a**) HO-1, (**b**) TNF-α and (**c**) IL-1β were determined by qRT-PCR. ***p < 0.001*.*

To confirm these results, Nrf2^-/-^ Raw 264.7 cells were engineered (Supplementary Fig. 6). While the expression of HO-1 and GPx-3 increased significantly in the control macrophages following infection with *S. mitis* or *S. oralis* (Fig. 6a, Supplementary Fig. 7), this change was not observed in Nrf2^-/-^ macrophages, providing evidence that these antioxidant genes were under the regulation of Nrf2. In Nrf2^-/-^ macrophages, treatment with *S. mitis* or *S. oralis* led to enhanced TNF-α and IL-1β levels compared to wild-type macrophages, demonstrating that activation of Nrf2 suppressed the innate immune response (Fig. 6b,c).

## Discussion

As periodontal health transitions to disease, a shift occurs in the oral microbiota. In disease, the amount of oral streptococci is diminished and the microbiota is dominated by gram-negative anaerobes^10,30–32^. A key metabolite secreted by oral streptococci species is H_2_O_2_. Previous studies have reported that H_2_O_2_ of oral streptococci led to the loss of viability of macrophages and oral epithelial cells^33–35^. However, those studies employed high bacterial loads and infection time. In this study, using bacterial loads which did not affect the viability of macrophages, we discovered that the production of H_2_O_2_ by oral streptococci inhibited the activation of NFĸB through the upregulation of the Nrf2-ARE antioxidant defence system pathway. Moreover, we showed that the SpxB was the bacterial effector involved in this process.

The ability to colonise and evade host defences would provide bacterial pathogens an advantage to survive. Since the NFĸB regulates immune surveillance, it is not surprising that successful pathogens have developed diverse strategies to evade host defences by exploiting or manipulating the NFĸB signalling pathway. For instance, the SseL of *Salmonella enterica* subsp. enterica serovar Typhimurium inhibits the ubiquitination of the inhibitory κB (IĸB) protein, preventing IĸB protein from degradation by the proteasome system^36,37^. Consequently, NFĸB remains sequestered in the cytoplasm, causing immune suppression. On the other hand, *S. pneumoniae* secreted H_2_O_2_ which inhibits the activation of inflammasomes through oxidative inactivation of the adaptor protein ASC and caspases^22^.

In this study, we discovered that *S. mitis* and *S. oralis* inhibited NFĸB in a H_2_O_2_ dependent manner. We further discovered that H_2_O_2_ secreted by *S. mitis* and *S. oralis* significantly downregulated LPS and *F. nucleatum*-mediated NFĸB activation. *F. nucleatum* is one of the early gram-negative bacterial species colonising the plaque biofilm. Compared to other gram-negative oral bacteria, we and others have demonstrated that *F. nucleatum* triggers strong pro-inflammatory responses^38–40^. Due to its ability to coaggregate with a wide variety of microorganisms, *F. nucleatum* plays an important role in plaque biofilm development serving as a key bridging organism between the early and late colonisers. The late colonisers, such as *P. gingivalis*, *Treponema denticola* and *Tannerella forsythia* are organisms associated with periodontal destruction^41^. Interestingly, in the absence of *F. nucleatum*, the amounts of periodontal disease associated bacteria reduced significantly^42,43^. Thus, through dampening of the immune response against *F. nucleatum* and LPS, it can be hypothesised that the SpxB of oral streptococci could potentially enhance the colonisation and survival of *F. nucleatum* and other gram-negative organisms in the plaque biofilm.

We found that in the presence of bacterial H_2_O_2,_ the Nrf2 pathway was activated, resulting in an elevation in HO-1 and GPx3. HO-1 is an antioxidant that protects multiple organs against oxidative stress and regulates a variety of cellular activities, including cell oxidation and apoptosis^44^. HO-1 is a rate-limiting enzyme in heme catabolism and is responsible for the degradation of pro-inflammatory free heme into anti-inflammatory compounds such as carbon monoxide, free iron and bilirubin, which play major roles in maintaining the protective effects of HO-1^45^. The critical role of Nrf2 mediated HO-1 expression for the anti-inflammatory activity was substantiated in a series of in vitro and in vivo experiments^45^. For instance, the elevation of Nrf2 mediated HO-1 expression leads to the inhibition of NFĸB signalling by preventing nuclear translocation of NFĸB, leading to a reduction in intestinal mucosal injury^46^. GPx3 is a selenium-containing antioxidant enzyme that reduces H_2_O_2_ and peroxide radicals with reduced glutathione (GSH), which is concomitantly oxidised to form oxidised glutathione (GSSG)^47,48^. As the major small molecule thiol in most organisms, GSH functions as a co-factor for GPx enzymes that quench H_2_O_2_ and other ROS^49,50^. Acute and chronic inflammation in the mice model has been shown to be caused by low GPx enzymatic activity in the mucosal epithelial^49,51,52^. In addition, increased GPx activity is associated with increased microbicidal activity in neutrophils, suggesting that GPx has potential anti-inflammatory properties^53,54^.

Studies have shown that H_2_O_2_ elicits activation of Nrf2 during oxidative stress^55,56^. Yet, it has been reported that the protein expression level of Nrf2 does not differ between periodontitis and periodontally healthy tissues^57^. The physiologically and clinical implications of Nrf2 activation by H_2_O_2_ producing oral streptococci are likely important during the early stages of colonisation of plaque biofilm formation. The ability to subvert the host's innate immune response will likely enable the bacteria with a colonisation advantage. However, as the plaque biofilm matures, the amount of oxygen available will decrease since bacteria within the biofilm matrix consume oxygen as they metabolise dietary sugars^58,59^. It has been shown that after 2 days of in vitro culture of plaque biofilm, the amount of dissolved oxygen content at the base of the biofilm reduced from 6 to 0.1 mg/l^59^. Additionally, a study also reported that the average oxygen profile measured *in* situ on dental biofilms decreased from 40 to 0% as the thickness of the biofilm increased^60^. Since the production of H_2_O_2_ by pyruvate oxidase is an oxygen-dependent process it can be anticipated that the H_2_O_2_ production by oral streptococci will decrease as plaque biofilm matures. This may explain why it was immunohistochemically demonstrated that the protein expression level of Nrf2 does not differ between periodontitis and periodontally healthy tissues, since the production of H_2_O_2_ by oral streptococci is expected to be low in these mature plaque biofilm^57^.

In summary, our results suggest that oral streptococci are likely not passive bystanders but could play an essential role in the maintenance of periodontal health through the prevention of overt inflammation. Future work will be required to understand the regulatory mechanisms involved in H_2_O_2_ production by oral streptococci, which might enhance our understanding of periodontal disease development. Since oral commensals naturally employ the Nrf2 pathway to downregulate inflammatory responses, another interesting area of further research will be to explore and identify suitable Nrf2 pathway modulators in the design of the next generation oral health care products for the prevention and treatment of oral inflammatory diseases.

## Methods

### Bacterial strains and culture

*Streptococcus mitis* ATCC 49456, *Streptococcus oralis* ATCC 35037, and *Streptococcus mutans* UA159 were obtained from the American Type Culture Collection (Manassas, VA, USA). Bacteria were cultured in brain heart infusion (BHI) broth (Acumedia, Lansing, MI, USA), and incubated at 37 °C with 5% CO_2_ supplementation.

### Prussian blue agar assay

The Prussian Blue Agar was employed to detect H_2_O_2_ producing oral streptococci. This agar comprised 52 g/L of BHI agar (Acumedia), 1 g/L of iron (III) chloride (Sigma-Aldrich, St. Louis, MO, USA) and 1 g/L of potassium hexacyanoferrate (Sigma). Overnight cultures of oral streptococci were spotted on Prussian Blue agar, and incubated at 37 °C for 4 h before visualization.

### spxB mutant generation and complementation

The *spxB* deletion (*ΔspxB*) mutants of *S. mitis* and *S. oralis* were generated as described previously with modifications^33^. Genomic DNA of *S. mitis* and *S. oralis* were extracted from overnight culture using QIAamp Mini kit (Qiagen, CA, USA). The upstream and downstream regions of the *spxB* gene were amplified using the primers spxKO-F1-BamHI and spxKO-R1. The PCR reactions consisted of 100 ng genomic DNA, 12.5 µl GoTaq PCR Master Mix (Promega, Madison, WI, USA), 0.2 µM of forward and reverse primers in a final reaction volume of 25 µl. The sequences of the primers used in the mutagenesis process are listed in Table 1. An aliquot of the purified upstream and downstream PCR products was used as a template in subsequent PCR reactions with the primer pair spxKO-F1-BamHI and spxKO-R2-SacI to yield an overlapping PCR fragment. The PCR products were purified and digested with *Bam*HI and *Sac*I, and ligated to *Bam*HI and *Sac*I digested pSET4s plasmid (Life Science Market, Scientific Hub Services, Singapore). Competent *S. mitis* and *S. oralis* were prepared by inoculating overnight bacterial culture into BHI media until OD_600_ of 0.05 was achieved. These bacterial cultures were incubated at 37 °C and 5% CO_2_ and growth were monitored every hour until OD_600_ reached 0.5. The bacterial cells were centrifuged at 3200 × *g* at 4 °C for 30 min, and the resulting supernatant was discarded. Bacterial cells were washed thrice with 10% ice-cold glycerol and resuspended in electroporation buffer (5 mM potassium phosphate [pH 4.5], 0.4 M sorbitol, 10% glycerol), and stored at − 70 °C.

**Table 1:** Sequences of oligonucleotides used in mutagenesis.

Primer	Sequence (5′ to 3′)	References
SpxKO-F1-BamHI	ATGGATCCCATCTTTATTATAAGCACCCTCAC	^33^
SpxKO-R1	GAGAGTTATCATTATGACTCAGGATTGCAATC ACGCGCAATC	^33^
SpxKO-F2	TTGCGCGTGATTGCAATCCTGAGTCATAATGA TAACTCTCCTTC	^33^
SpxKO-R2-SacI	GCGAGCTCATTGGTATCGAAGAGGTCATTGC	This study
Spx-inside-F	ACCATGGAGTAGACTCAACTGG	^33^
Spx-inside-R	ATGGATAACACTCCATTCCTTG	^33^
SpxB-EcoRI-F	ATGAATCCGGATTGCTCCGATCTTTTCA	This study
SpxB-BamHI-R	CTGGATCCCGAGCTTGCTCGCTTTTTAT	This study

The recombinant plasmids were electroporated into competent *S. mitis* and *S. oralis* using a Bio-Rad MicroPulser™ electroporator (Bio-Rad Laboratories) at 3.0 kV using 0.2 cm electroporation cuvette (Bio-Rad Laboratories, Hercules, CA, US). Transformants were grown at 28 °C and selected on BHI agar plates containing spectinomycin (100 µg/ml). Single-crossover mutants were selected by culturing the bacteria on agar plates containing spectinomycin at 37 °C. Double-crossover mutants were obtained by repeated passaging on agar plates with no antibiotics at 28 °C, and determined by PCR using the primers targeting an internal region of the *spxB* gene (i.e. spx-inside-F and spx-inside-R); and subsequently confirmed by DNA sequencing.

*S. mitis* and *S. oralis spxB* mutants with *spxB* gene complementation were engineered using the method described previously^61^. The full length *spxB* gene was obtained by PCR using the primers SpxB-EcoRI-F and SpxB-BamHI-R (Table 1), was cloned into the *Streptococcus-E. coli* shuttle plasmid pDL278 via the *Eco*RI and *Bam*HI restriction sites. The recombinant plasmid was introduced into the *spxB* mutants by electroporation and selected on agar plates containing spectinomycin as described above.

### H_2_O_2_ assay

The amount of H_2_O_2_ produced by wild-type and *ΔspxB S. mitis and S. oralis* were determined using the ROS-Glo™ H_2_O_2_ Assay Kit (Promega) according to the manufacturer’s instruction.

### Cell culture

The Raw 264.7 macrophage cell line was obtained from the ATCC. Cells were cultured in DMEM (Hyclone) supplemented with 10% FBS (Hyclone). The Raw 264.7 macrophage stably expressing the NFĸB-SEAP reporter^62^ was cultured in DMEM + 10% FBS supplemented with 500 μg/ml geneticin (Sigma-Aldrich). Bone marrow derived macrophages (BMDMs) were obtained from Cell Biologics (Cell Biologics, Chicago, USA) and cultured as described previously^63^. All cells were incubated at 37 °C in a humidified incubator supplemented with 5% CO_2_.

### Generation of macrophage stable cell line stably expressing the ARE-SEAP reporter

An antioxidant response element (ARE)-SEAP reporter vector was generated by annealing two phosphorylated oligonucleotides with the ARE motif (ARE-F and ARE-R, Table 2). The resulting double-stranded fragment was subsequently ligated into *Kpn*I and *Bgl*II digested pNFĸB-SEAP reporter plasmid expressing the neomycin gene resistance cassette, which encodes resistance to geneticin^62^. The sequence of the recombinant vector engineered was confirmed by DNA sequencing. Raw 264.7 macrophages were transfected with the ARE-SEAP reporter plasmid using Lipofectamine 3000 (Thermo Fisher Scientific, Waltham, MA, US) according to the manufacturer’s protocol. SEAP activity in the culture supernatant was measured using a Phospha-Light assay kit (Life Technologies, Rockville, MD, USA), according to the manufacturer’s protocol.

**Table 2:** ARE oligonucleotide sequences used in this study.

Oligonucleotide	Sequence (5′ to 3′)	References
ARE-F	CTAGCTTGGAAATGACATTGCTAATGGTGACAAAGCAACTTTTAGCTTGGAAATGACATTGCTAATGGTGACAAAGCAACTTTA	This study
ARE-R	GATCTAAAGTTGCTTTGTCACCATTAGCAATGTCATTTCCAAGCTAAAAGTTGCTTTGTCACCATTAGCAATGTCATTTCCAAGCTAGGTAC	This study

### Cell infection

Cells were infected with oral streptococci at the required multiplicity of infection (MOI), in the presence or absence of 25U/ml of catalase (Sigma-Aldrich) and incubated at 37 °C in a humidified incubator supplemented with 5% CO_2_ for 8 h. Cellular ARE and NFĸB activities were quantified using the SEAP reporter assay as described above.

### Cell viability assays

The viability of macrophages after bacterial infection were determined by trypan blue exclusion and Lactate Dehydrogenase (LDH) assays. For trypan blue assay, equal volume of 0.4% of trypan blue (Sigma-Aldrich) was added to cell suspension and the number of viable cells enumerated. The LDH assay was carried out using the CytoTox 96 Non-Radioactive Cytotoxicity Assay Kit (Promega) according to the manufacturer’s protocol.

### Immunofluorescence

Raw 264.7 cells were seeded at a density of 3 × 10^4^ cells/well in a 96-well plate. BMDMs were seeded at a density of 1 × 10^4^ cell/well. These cells were infected with oral streptococci as described above. Cells were fixed with 4% paraformaldehyde, followed by permeabilization with 0.2% Triton-X in PBS for 15 min and blocked for 1 h at room temperature with PBS containing 5% bovine serum albumin (Sigma-Aldrich). Cells were stained with primary antibody against Nrf2 (ab31163; Abcam, MA, USA) overnight at 4 °C. Subsequently, cells were incubated with goat anti-rabbit IgG (Alexa Fluor® 488, ab150077; Abcam) in the dark for 1 h at room temperature. Nuclei and cytoskeleton were sequentially counterstained with 4′6-diamidino-2-phenylindole, DAPI (Abcam) and Phalloidin-iFluor 594 reagent (Abcam) respectively. Representative fluorescence images were captured using a DMi8 fluorescence microscope (Leica Microsystems, Wetzlar, Germany).

### ML385 treatment

Raw 264.7 macrophages were seeded at a density of 3 × 10^4^/well in 96-well tissue culture plates (Nunc) and allowed to adhere overnight. The Nrf2 inhibitor ML385 (Sigma) was added into the cell culture media at 10 µM and incubated for 24 h. The next day, cells were infected with oral streptococci for 8 h. Culture supernatant was harvested and the amount of cellular ARE or NFĸB activity was quantified by measuring SEAP reporter activity as described above. The expression of target genes was determined by qPCR.

### Nrf2 gene knockout

Raw 264.7 cells with Nrf2 gene knockout were engineered via the CRISPR/Cas9 genome editing method. pLentiCRISPRv2 plasmids harbouring the Nrf2 CRISPR gRNA target sequences 5′-TAGTTGTAACTGAGCGAAAA (NFE2L2 CRISPR Guide RNA 1) and 5′-GCATACCGTCTAAAT CAAC (NFE2L2 CRISPR Guide RNA 2) were obtained from GenScript (NJ, USA). The pLentiCRISPRv2 plasmids were transfected into Raw 264.7 cells using Lipofectamine 3000 (ThermoFisher Scientific). The efficiency of Nrf2 knockdown was determined via immunofluorescence staining with primary antibodies against Nrf2 as described above.

### RNA extraction and qPCR analysis

Total RNA from treatment and non-treatment groups of Raw 264.7 cells were extracted using the RNeasy Mini Kit (Qiagen, Valencia, CA, USA) with on-column DNaseI treatment according to the manufacturer’s protocol. Reverse transcription (RT) of RNA to cDNA was performed using the iScript Reverse Transcription SuperMix (BioRad, Hercules, CA, USA). qPCR was performed using a CFX Connect Real-Time Detection System (BioRad). The reaction mixture consisted of 1 μl of cDNA, 10 μl of iTaq Universal SYBR Green Supermix (BioRad), 1 μl each of 10 µM of respective forward and reverse primers and nuclease-free water in a final volume of 20 μl. The expression of the respective target gene was normalized to the relative abundance of a housekeeping gene, beta actin. Fold changes in gene expression were calculated using the ^ΔΔ^CT method. The sequences of primers used are listed in Table 3.

**Table 3:** Primer sequences used for qPCR analysis.

Primer	Sequence (5′ to 3′)	References
GPx-3-F	ACAATTGTCCCAGTGTGTGCAT	This study
GPx-3-R	TGGACCATCCCTGGGTTTC	This study
HO-1-F	AAGCCGAGAATGCTGAGTTCA	This study
HO-1-R	GCCGTGTAGATATGGTACAAGGA	This study
TNF-α-F	CATCTTCTCAAAATTCGAGTGACCA	This study
TNF-α-R	TGGGAGTAGACAAGGTACAACCC	This study
IL-1β-F	CAACCAACAAGTGATATTCTCCATG	This study
IL-1β-R	GATCCACACTGTCCAGCTGCA	This study
Beta actin-F	GGCTGTATTCCCCTCCATCG	This study
Beta actin-R	CCAGTTGGTAACAATGCCATGT	This study

### Enzyme-linked immunosorbent assay (ELISA)

The amount of TNF-α and IL-1β protein in the culture supernatant were quantified by ELISA using the ELISA kits from Biolegend (San Diego, CA, USA). ELISA was performed according to the manufacturer’s instruction.

### Statistical analysis

All experiments were performed in triplicate and repeated 3 independent times. The values were expressed as the mean ± standard deviation. Statistical analyses were performed using GraphPad Prism 8 software (GraphPad Software, Inc., USA). Differences between groups were assessed using one-way analysis of variance followed by Tukey's post hoc multiple comparison test. *p* < 0.05 was considered to indicate a statistically significant difference.

## Supplementary Information

Supplementary Information.
