# Supplementary material for: Oral streptococci subvert the host innate immune response through hydrogen peroxide
Source: Sci Rep. 2022 Jan 13;12:656. doi: 10.1038/s41598-021-04562-4 (PMC8758666; doi:10.1038/s41598-021-04562-4)
Supplement: Supplementary file 1 — Supplementary Information. [file 41598_2021_4562_MOESM1_ESM.docx]

**Oral streptococci subvert the host innate immune response through hydrogen peroxide**

Yi Ling Tang^1^, Tiow Suan Sim^2^ and Kai Soo Tan^1*^

^1^Faculty of Dentistry, National University of Singapore, Singapore

^2^ Department of Microbiology and Immunology, Yong Loo Lin School of Medicine,

National University of Singapore, Singapore

^*^Corresponding author:

Kai Soo Tan

Faculty of Dentistry

National University of Singapore

9 Lower Kent Ridge Road,

National University Centre for Oral Health

Singapore 119085

Email: [denkst@nus.edu.sg](mailto:denkst@nus.edu.sg)

Tel: +65-6772-8842

Fax: +65-6778-5742

**Supplementary Fig. 1**

**
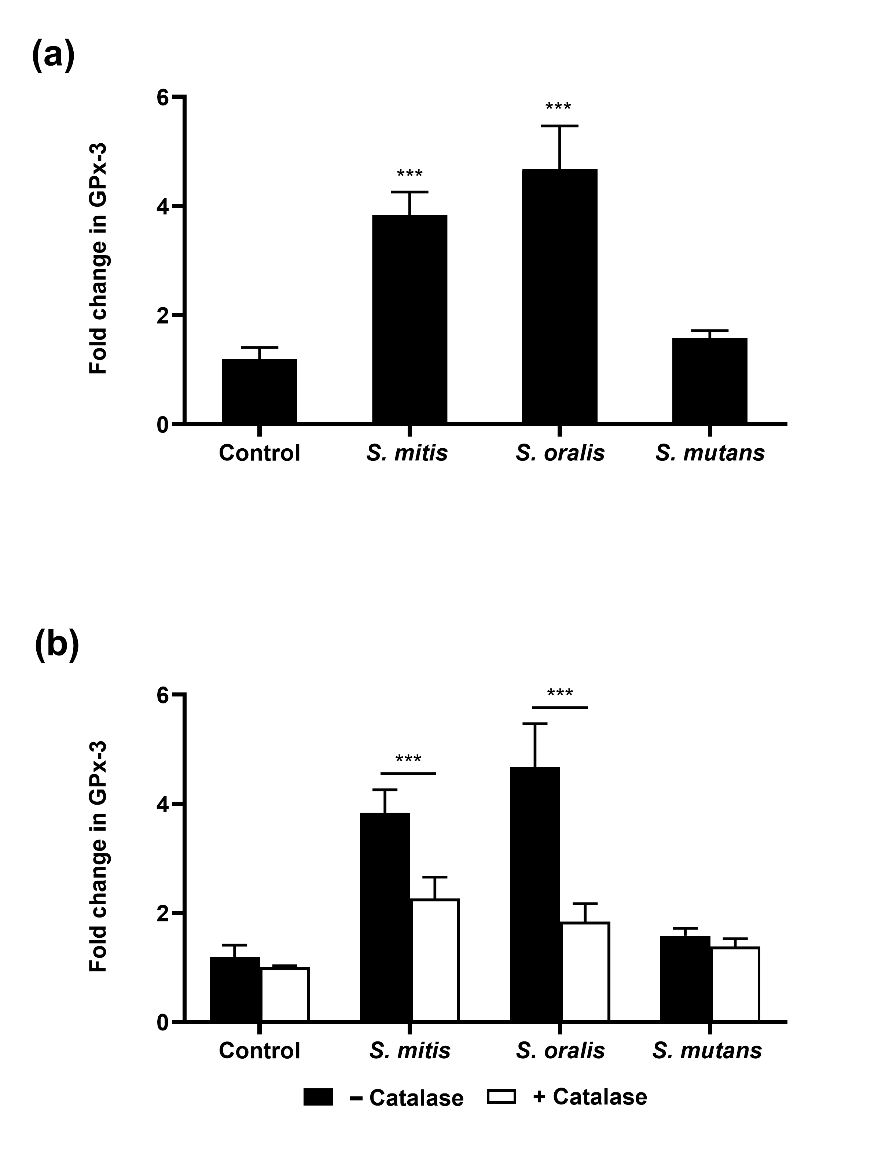
**

Supplementary Fig. 1. H_2_O_2_ producing oral streptococci up-regulated the expression of GPx-3 in macrophages. (a) Raw 264.7 cells were infected with *S. mitis, S. oralis* or *S. mutans* at MOI 50:1 and the expression of GPx-3 was determined by qRT-PCR. (b) Raw 264.7 cells were infected with *S. mitis* or *S. oralis* at MOI 50:1 in the presence and absence of 25U/mL of catalase and the expression of GPx-3 was determined by qRT-PCR. ****p<0.001*

**Supplementary Fig. 2**


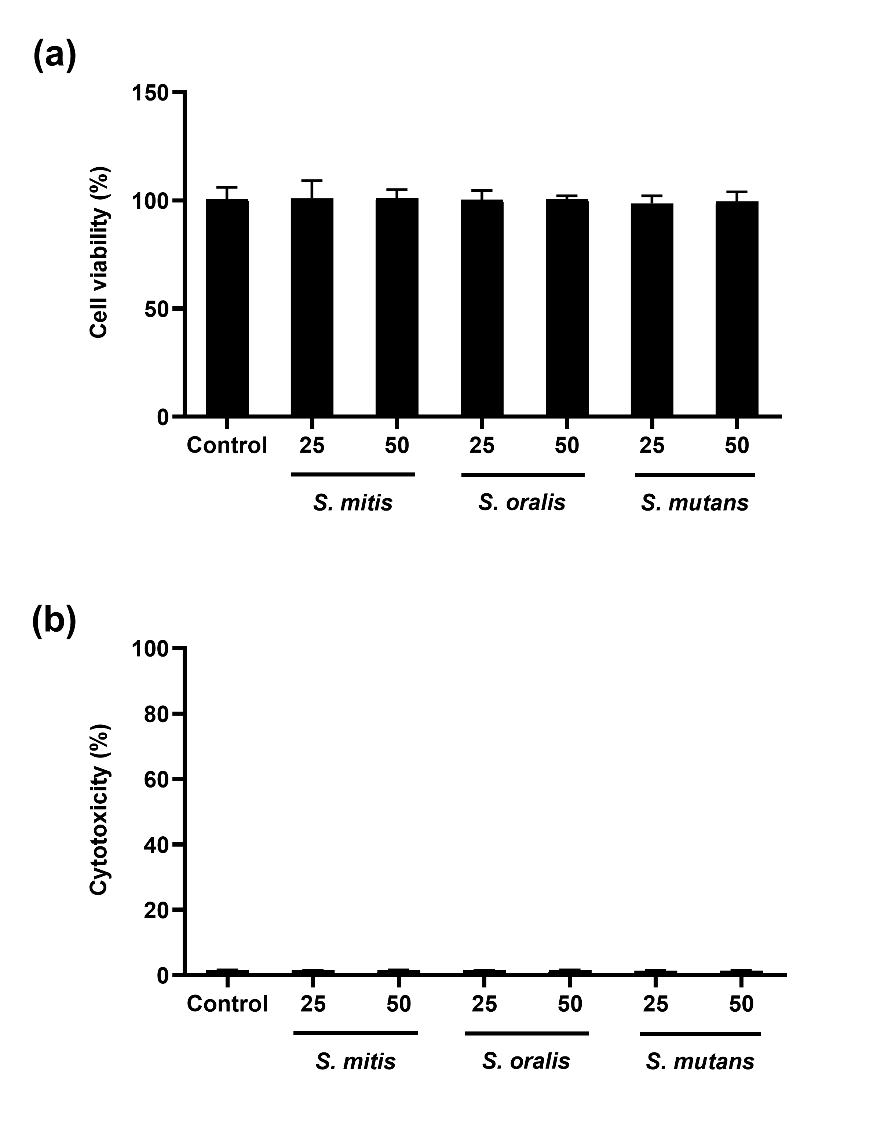


Supplementary Fig. 2. Viability of macrophages following infection with oral streptococci. Raw 264.7 cells were infected with the indicated species and MOI of oral streptococci for 8h. The viability of macrophages was determined by (a) trypan blue and (b) LDH assays.

**Supplementary Fig. 3**

**
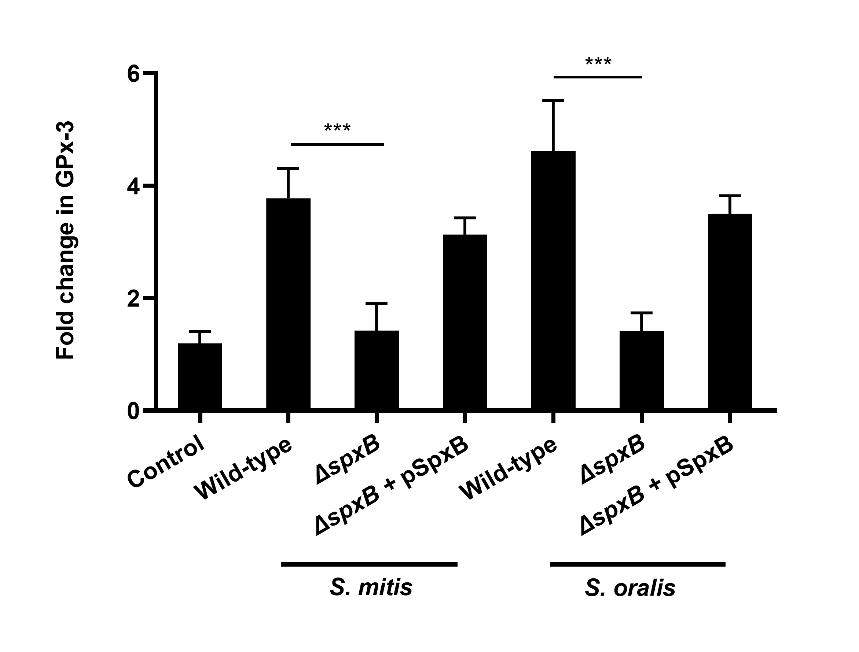
**

Supplementary Fig. 3. Bacterial SpxB mediated the activation of the antioxidant gene GPx-3. Raw 264.7 cells were infected with wild-type, *ΔspxB* mutant or complemented mutant (*ΔspxB* + pSpxB) of *S*. *mitis* or *S. oralis*. The expression of GPx-3 was determined by qRT-PCR. ****p<0.001*

**Supplementary Fig. 4**

**
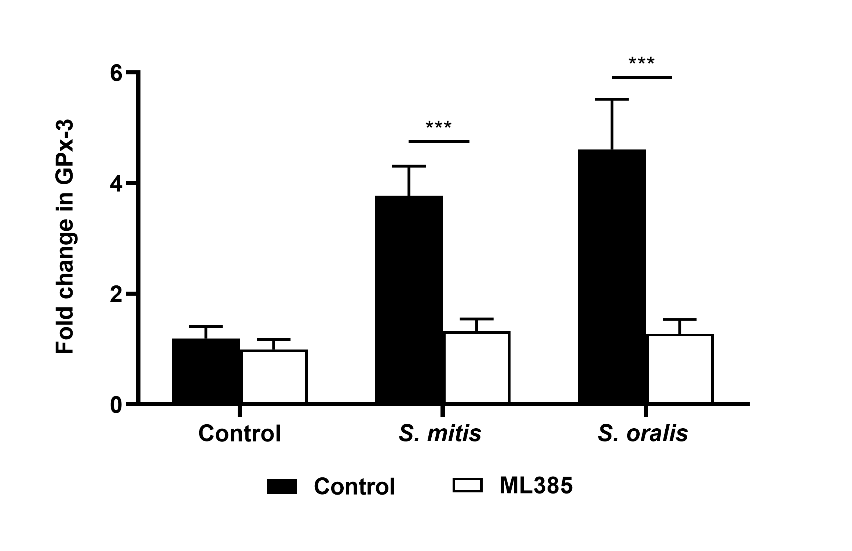
**

Supplementary Fig. 4. Activation of Nrf2 pathway by oral streptococci elicited GPx-3 expression. Raw 264.7 cells were pre-treated with 10µM ML385 for 24h prior to infection with oral streptococci. The expression of GPx-3 was determined by qRT-PCR. ****p<0.001*

**Supplementary Fig. 5**

**
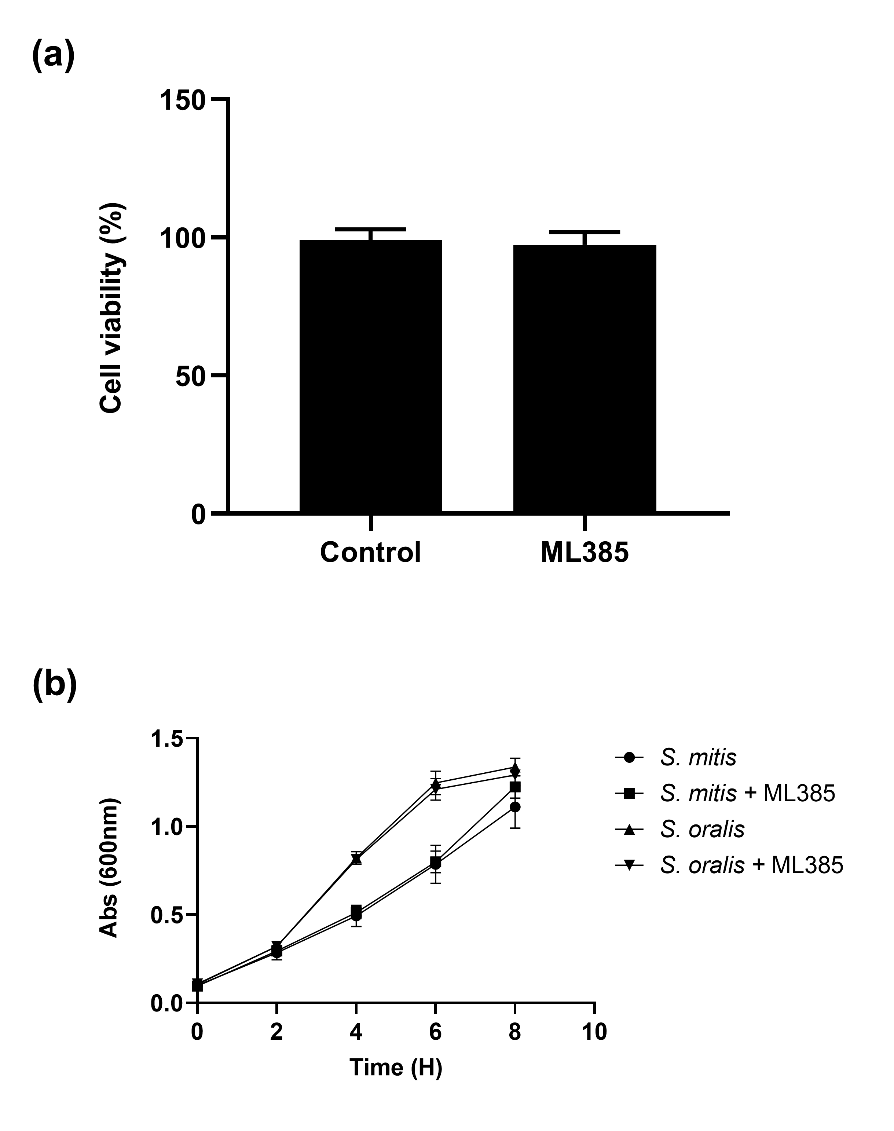
**

Supplementary Fig. 5. Effects of ML385 on viability of macrophages and oral streptococci. Raw 264.7 cells were treated with 10µM of ML385 for 32h and (a) the viability of macrophages was determined by trypan blue. (b) The viability of the oral streptococci over the duration of 8h in the presence of 10µM of ML385 were determined by measuring the absorbance at OD_600_.

**Supplementary Fig. 6**


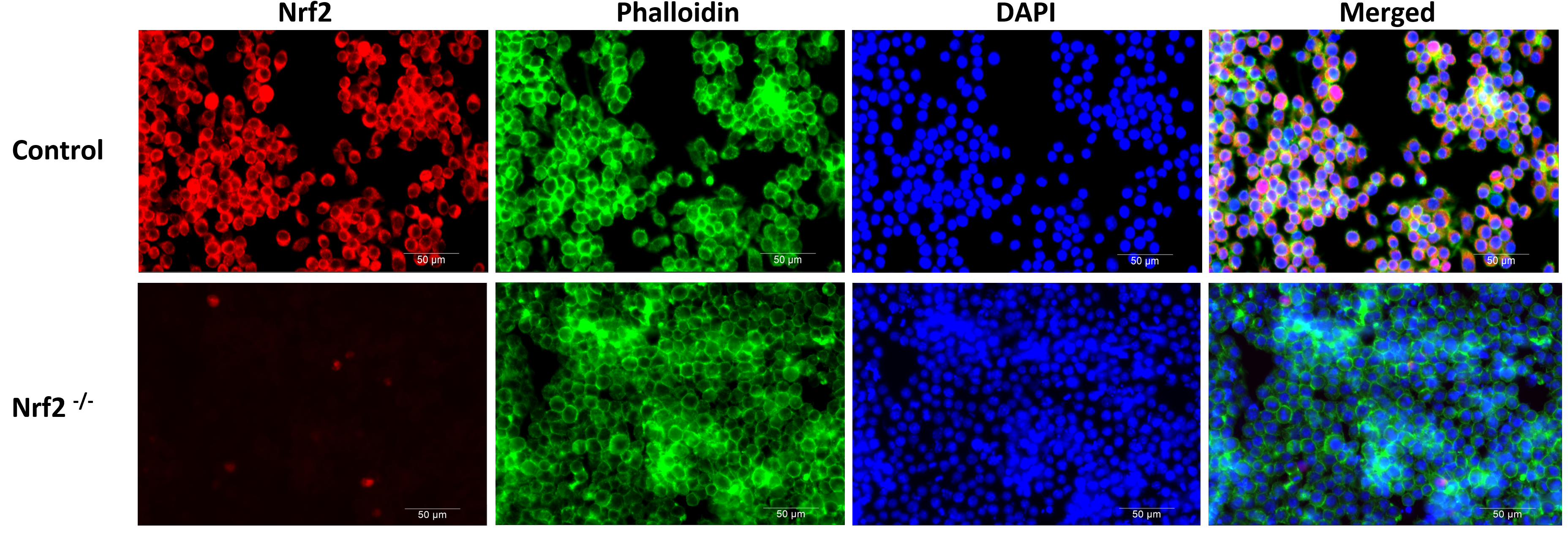


Supplementary Fig. 6. Expression of Nrf2 in control (wild-type) and Nrf2^-/-^ macrophages. Raw 264.7 cells were transfected with pLentiCRISPR plasmid expressing Nrf2 gRNA. Nrf2 was stained red, DAPI stained the nucleus blue while phalloidin stained the actin green. Representative immunofluorescence images at 400x magnification. Scale bar: 50µm

**Supplementary Fig. 7**

**
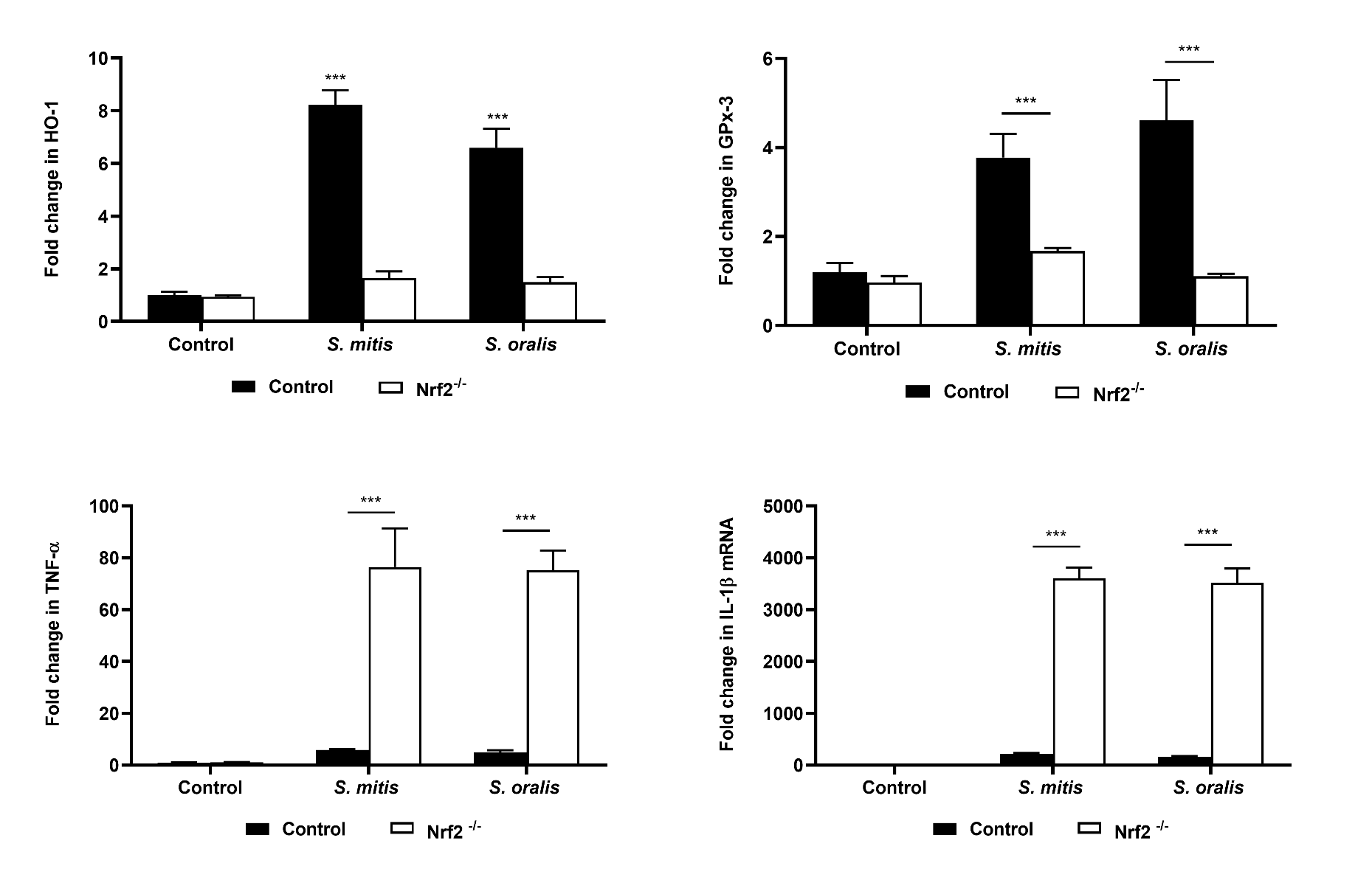
**

Supplementary Fig. 7. Oral streptococci mediate the expression of GPx-3 through Nrf2. Wild-type (control) or Nrf2^-/-^ Raw 264.7 cells were infected with oral streptococci for 8h and the expression of GPx-3 was determined by qRT-PCR. ****p<0.001*
